# Supplementary material for: Effect and Mechanisms of Antibacterial Peptide Fraction from Mucus of C. aspersum against Escherichia coli NBIMCC 8785
Source: Biomedicines. 2022 Mar 14;10(3):672. doi: 10.3390/biomedicines10030672 (PMC8945727; doi:10.3390/biomedicines10030672)
Supplement: Supplementary file 1 [file biomedicines-10-00672-s001.zip › biomedicines-1620739-supplementary.pdf]

Supplementary Information (SI) File S1. Digital image analysis of the fluorescence images of *E. coli* after 6-hours incubation with peptides fraction with MW below 10 kDa.

Peptides Fraction	Peptides Concentration	Fluorescence Intensity (CTC)	Cells Mean Perimeter, μm (DAPI)	Circularity (DAPI)	Cells Area, μm²/Cell (DAPI)	Live/Dead Cells Ratio (CTC/DAPI), %
Control	-	219.00 ±37.80	9.87 ±2.15	0.519 ±0.016	3.80 ±1.43	92.51% ±19.87%
Peptides fraction with MW below 10 kDa	1%	227.60 ±6.35	9.96 ±2.45	0.470 ±0.036	3.29 ±1.10	87.82% ±10.65%
	5%	220.00 ±2.31	9.25 ±0.54	0.479 ±0.021	3.01 ±0.27	77.07% ±9.12%
	10%	198.20 ±15.02	10.78 ±1.64	0.484 ±0.021	4.07 ±1.12	95.24% ±12.85%

Supplementary Information (SI) File S2

An alignment of amino acid sequences of some identified peptides from *C. Aspersum* mucus with known antimicrobial peptides with data base AMPs by CAMPSing (**http://www.campsign.bicnirrh.res.in/blast.php**) and with proteins by BLAST (**https://blast.ncbi.nlm.nih.gov**).

PEPTIDE 1. AAGLAGAGGGGGG

```
> APD_2350|AP02350|Microcin B
Length=42, Score = 25.0 bits (49),Expect = 0.028, Identities = 8/11 (73%),
Positives = 9/11 (82%), Gaps = 0/11 (0%)
P 1    3   GLAGAGGGGGG  13
           G+ G GGGGGG
Sbjct  2   GIGGGGGGGGG  12
```

> APD_255|AP01227|Microcin B17 from *Escherichia coli*

```
Length=43, Score = 25.0 bits (49, Expect = 0.030, Identities = 8/11 (73%),
Positives = 9/11 (82%), Gaps = 0/11 (0%)
P 1    3   GLAGAGGGGGG  13
           G+ G GGGGGG
Sbjct  2   GIGGGGGGGGG  12
```

> CAMPSQ3894,Leptoglycin from *Leptodactylus pentadactylus*, Antibacterial(Gram-)

```
Length=22; Score = 23.7 bits (46,Expect = 0.054; Identities = 8/10 (80%),
Positives = 8/10 (80%), Gaps = 0/10 (0%)
P 1    4   LAGAGGGGGG  13
           L G GGGGGG
Sbjct  11  LGGGGGGGGG  20
```

PEPTIDE 2. DKGLGGFEA

```
RecName: Full=Hemocyanin A-type, units Ode to Odg [Enteroctopus dofleini]
```

Sequence ID: P12659.2, Length: 1233, Score16.3 bits(31) Expect = 6.9, Identities=6/9(67%), Positives=6/9(66%) Gaps0/9(0%)

```
P 2    1    DKGLGGFEA  9
            DK   GFEA
Sbjct  32   DKSSDGFEA  40
```

PEPTIDE 3. LGDLNAEFAAG

RecName: **Partition protein-ADP complex [Escherichia coli]**
Sequence ID: 3EZ2_A, Length:398,Score=25.2 bits(52), Expect=1.6, Identities=7/8(88%), Positives=7/8(87%), Gaps=0/8(0%)

```
Query  8    ANPSTYVG  15
            ANP TYVG
Sbjct  362  ANPATYVG  369
```

PEPTIDE 4. AGVGAGGANPSTYVG

> CAMPSQ7361, osmotin-like protein-like from *Fragaria vesca subsp*. vesca Length=248,Score = 21.9 bits (42),Expect = 0.94,Identities = 7/9 (78%), Positives = 8/9 (89%), Gaps = 0/9 (0%)

```
P 4    4    GAGGANPST  12
            GAGGA P+T
Sbjct  107  GAGGATPAT  115
```

PEPTIDE 5. GAACNLEDGSCLGV

> CAMPSQ932, Defensin J1-1 from Capsicum annuum [Bell pepper], Antifungal Length=48;Score = 18.9 bits (35), Expect = 6.0,Identities = 8/16 (50%), Positives = 9/16 (56%), Gaps = 3/16 (19%)

```
P 5    1    GAACNLE---DGSCLG  13
            G  C  E   DGSC+G
Sbjct  21   GNVCRREGFTDGSCIG  36
```

PEPTIDE 6. EPGGGGGEGGGLLGVAL

> CAMPSQ3894, Leptoglycin from *Leptodactylus pentadactylus*,Antibacterial(Gram-)
Length=22,Score = 26.7 bits (53), Expect = 0.006, Identities = 10/11 (91%), Positives = 10/11 (91%), Gaps = 0/11 (0%)

```
P 6    3    GGGGGEGGGLL  13
            GGGGG GGGLL
Sbjct  12   GGGGGGGGGLL  22
```

> CAMPSQ3973, Glycine-rich protein GWK from *Cucumis melo* [Muskmelon], Antifungal
Length=36, Score = 25.0 bits (49), Expect = 0.031,Identities = 8/9 (89%), Positives = 9/9 (100%), Gaps = 0/9 (0%)

```
P 6    3    GGGGGEGGG  11
            GGGGG+GGG
Sbjct  27   GGGGGKGGG  35
```

PEPTIDE 7. LGPLYDEMGPVGGDVG

> CAMPSQ5771, Zeamatin from *Cucumis melo* subsp. melo, Antimicrobial
Length=156, Score = 19.8 bits (37), Expect = 4.5,Identities = 6/10 (60%), Positives = 7/10 (70%), Gaps = 0/10 (0%)

```
P 7    7    EMGPVGGDVG  16
            EM P+GG  G
Sbjct  138  EMAPIGGGSG  147
```

PEPTIDE 8. ASKGCGPGSCPPGDTVAGVG

> CAMPSQ2192, Crustin-like antimicrobial peptide from *Fenneropenaeus indicus* [Indian white prawn], Antimicrobial

Length=117,Score = 25.0 bits (49),Expect = 0.11, Identities = 9/16 (56%), Positives = 10/16 (63%), Gaps = 1/16 (6%)

```
P 8    5   CGPGSCPP-GDTVAGV  19
           C PG CPP  DT  G+
Sbjct  63  CKPGRCPPVRDTCPGI  78
```

> CAMPSQ3075, Gallinacin 8 from *Gallus gallus* [Chicken]
Length=41,Score = 23.7 bits (46),Expect = 0.14,Identities = 7/14 (50%),Positives = 10/14 (71%), Gaps = 0/14 (0%)

```
P 8    5   CGPGSCPPGDTVAG  18
           C  G+CPP  T++G
Sbjct  12  CRAGACPPTFTISG  25
```

> CAMPSQ1841 Gallinacin 10 from Gallus gallus [Chicken]
Length=43,Score = 23.7 bits (46), Expect = 0.15,Identities = 7/14 (50%), Positives = 10/14 (71%), Gaps = 0/14 (0%)

```
P 8    5   CGPGSCPPGDTVAG  18
           C  G+CPP  T++G
Sbjct  14  CRAGACPPTFTISG  27
```

PEPTIDE 9. ACSLLLGGGGVGGGKGGGGHAG

> CAMPSQ3913, Ctenidin-1 from *Cupiennius salei* [Wandering spider], Antimicrobial
Length=119,Score = 37.0 bits (77),Expect = 9e-006,Identities = 12/16 (75%), Positives = 15/16 (94%), Gaps = 0/16 (0%)

```
P 9    7    GGGGVGGGKGGGGHAG  22
            GGGG+GGG+GGGG+ G
Sbjct  88   GGGGLGGGRGGGGYGG  103
```

> CAMPSQ3914, Ctenidin-3 from *Cupiennius salei* [Wandering spider], Antimicrobial
Length=120,Score = 37.0 bits (77),Expect = 1e-005,Identities = 12/16 (75%), Positives = 15/16 (94%), Gaps = 0/16 (0%)

```
P 9    7    GGGGVGGGKGGGGHAG  22
            GGGG+GGG+GGGG+ G
Sbjct  90   GGGGLGGGRGGGGYGG  105
```

> DBAASP_2873|3113|Acanthoscurrin-2 from *Acanthoscurria gomesiana* [Tarantula spider], Antibacterial (Gram-), Antifungal, Target: *E.coli* SBS363, *C.albicans*
Length=130,Score = 33.1 bits (68),Expect = 2e-004, Identities = 13/20 (65%), Positives = 16/20 (80%), Gaps = 3/20 (15%)

```
P 9    6    LGGGGVGGGK---GGGGHAG  22
            LGGGG+GGG+   GGGG+ G
Sbjct  99   LGGGGLGGGRGGYGGGGYGG  118
```

> CAMPSQ1508, Acanthoscurrin-1 from Acanthoscurria gomesiana [Tarantula spider], Antibacterial (Gram-), Antifungal, Target: *E.coli* SBS363, *C.albicans*
Length=132,Score = 32.7 bits (67),Expect = 3e-004,Identities = 14/19 (74%), Positives = 15/19 (79%), Gaps = 2/19 (11%)

```
P 9    6   LGGGGVGGGK--GGGGHAG  22
           LGGGG+GGGK  GGGG  G
Sbjct  31  LGGGGLGGGKGLGGGGLGG  49
```

PEPTIDE 10. ACLTPVDHFFAGMPCGGGP

> CAMPSQ5060, Cyclotide 2a from *Viola baoshanensis,* Antimicrobial
Length=113,Score = 20.2 bits (38), Expect = 3.6,Identities = 8/16 (50%), Positives = 10/16 (63%), Gaps = 0/16 (0%)

```
P 10   1   ACLTPVDHFFAGMPCG  16
           A L  +DH   G+PCG
```

```
Sbjct  69  ALLKNLDHGRNGVPCG  84
```

PEPTIDE 11. NGLFGGLGGGGHGGGGKGPGEGGG

> CAMPSQ322, Holotricin-3 from *Holotrichia diomphalia* [Korean black chafer], Antibacterial, Antifungal

Length=84,Score = 39.2 bits (82),Expect = 1e-006,Identities = 16/23 (70%), Positives = 17/23 (74%), Gaps = 0/23 (0%)

```
P 11   2   GLFGGLGGGGHGGGGKGPGEGGG  24
           G FGG  GGGHGGGG+G G  GG
Sbjct  33  GGFGGGHGGGHGGGGRGGGGSGG  55
```

> CAMPSQ3914, Ctenidin-3 from *Cupiennius salei* [Wandering spider], Antimicrobial

Length=120,Score = 38.8 bits (81),Expect = 3e-006,Identities = 15/20 (75%), Positives = 16/20 (80%), Gaps = 0/20 (0%)

```
P 11   5   GGLGGGGHGGGGKGPGEGGG  24
           GG GGGG+GGGG G G GGG
Sbjct  11  GGYGGGGYGGGGGGYGGGGG  30
```

> CAMPSQ3913 Ctenidin-1 from *Cupiennius salei* [Wandering spider], Antimicrobial

Length=119, Score = 37.9 bits (79),Expect = 5e-006,Identities = 15/20 (75%), Positives = 16/20 (80%), Gaps = 0/20 (0%)

```
P 11   5   GGLGGGGHGGGGKGPGEGGG  24
           GGLGGG  GGGG G G+GGG
Sbjct  70  GGLGGGQGGGGGLGGGQGGG  89
```

> CAMPSQ1508, Acanthoscurrin-1 from *Acanthoscurria gomesiana* [Tarantula spider] Antibacterial (Gram-), Antifungal, Target: *E.coli* SBS363, *C.albicans*

Length=132,Score = 37.5 bits (78), Expect = 8e-006, Identities = 15/19 (79%), Positives = 15/19 (79%), Gaps = 0/19 (0%)

```
P 11   6    GLGGGGHGGGGKGPGEGGG  24
            GLGGGG GGGG G G GGG
Sbjct  93   GLGGGGLGGGGLGGGRGGG  111
```

PEPTIDE 12. LLLLMLGGGLVGGLLGGGGKGGG

>APD_453|AP01405|Leptoglycin from *Leptodactylus pentadactylus*,Antibacterial(Gram-)

Length=22,Score = 33.1 bits (68),Expect = 5e-005,Identities = 13/16 (81%), Positives = 14/16 (88%), Gaps = 0/16 (0%)

```
P 12   8   GGLVGGLLGGGGKGGG  23
           GGL+G LLGGGG GGG
Sbjct  4   GGLLGPLLGGGGGGGG  19
```

> APD_1344|AP02207|Acanthoscurrin 2 from Acanthoscurria gomesiana [Tarantula spider], Antibacterial (Gram-), Antifungal, Target: *E.coli* SBS363, *C.albicans*.

Length=131,Score = 33.6 bits (69),Expect = 2e-004,Identities = 14/17 (82%), Positives = 14/17 (82%), Gaps = 0/17 (0%)

```
P 12   7   GGGLVGGLLGGGGKGGG  23
           GGGL GG LGGGG GGG
Sbjct  23  GGGLGGGGLGGGGLGGG  39
```

Score = 33.6 bits (69), Expect = 2e-004,Identities = 14/17 (82%), Positives = 14/17 (82%), Gaps = 0/17 (0%)

```
P 12   7   GGGLVGGLLGGGGKGGG  23
           GGGL GG LGGGG GGG
Sbjct  44  GGGLGGGGLGGGGLGGG  60
```

> DBAASP_2872|3112|Acanthoscurrin-1 **from Acanthoscurria gomesiana [Tarantula spider], Antibacterial (Gram-), Antifungal, Target: *E.coli* SBS363, *C.albicans*.**
Length=132, Score = 33.6 bits (69), Expect = 2e-004,Identities = 14/17 (82%), Positives = 14/17 (82%), Gaps = 0/17 (0%)

```
P 12   7   GGGLVGGLLGGGGKGGG  23
           GGGL GG LGGGG GGG
Sbjct  23  GGGLGGGGLGGGGLGGG  39
```

PEPTIDE 13. PFLLGVGGLLGGSVGGGGGGGGAPL

> CAMPSQ3894,Leptoglycin from *Leptodactylus pentadactylus*,Antibacterial(Gram-)
Length=22,Score = 34.4 bits (71),Expect = 2e-005,Identities = 14/20 (70%), Positives = 16/20 (80%), Gaps = 0/20 (0%)

```
P 13   6   VGGLLGGSVGGGGGGGGAPL  25
           +GGLLG  +GGGGGGGG  L
Sbjct  3   LGGLLGPLLGGGGGGGGGLL  22
```

> CAMPSQ1805, Acanthoscurrin-2, from *Acanthoscurria gomesiana* [Tarantula spider], Antibacterial (Gram-), Antifungal, Target: E.coli SBS363, C.albicans
Length=131,Score = 33.1 bits (68),Expect = 3e-004,Identities = 14/19 (74%), Positives = 15/19 (79%), Gaps = 0/19 (0%)

```
P 13   4   LGVGGLLGGSVGGGGGGGG  22
           LG GGL GG +GGGG GGG
Sbjct  42  LGGGGLGGGGLGGGGLGGG  60
```

> DBAASP_2872|3112|Acanthoscurrin-1 from Acanthoscurria gomesiana [Tarantula spider], Antibacterial (Gram-), Antifungal, Target: *E.coli* SBS363, *C.albicans*
Length=132,Score = 33.1 bits (68),Expect = 3e-004,Identities = 14/19 (74%), Positives = 15/19 (79%), Gaps = 0/19 (0%)

```
P 13   4   LGVGGLLGGSVGGGGGGGG  22
           LG GGL GG +GGGG GGG
Sbjct  42  LGGGGLGGGGLGGGGLGGG  60
```

PEPTIDE 15. GLLGGGGGAGGGGLVGGLLNG

> APD_2350|AP02350|Microcin B
Length=42,Score = 31.0 bits (63), Expect = 3e-004,Identities = 11/15 (73%), Positives = 12/15 (80%), Gaps = 0/15 (0%)

```
P 15   3   LGGGGGAGGGGLVGG  17
           +GGGGG GGGG  GG
Sbjct  3   IGGGGGGGGGGSCGG  17
```

Score = 27.5 bits (55),Expect = 0.005,Identities = 14/27 (52%), Positives = 15/27 (56%), Gaps = 6/27 (22%)

```
P 15   1   GLLGGGGGAGG------GGLVGGLLNG  21
           G+ GGGGG GG      GG  GG  NG
Sbjct  2   GIGGGGGGGGGSCGGQGGGCGGCSNG  28
```

> APD_255|AP01227|Microcin B17
Length=43, Score = 31.0 bits (63),Expect = 3e-004,Identities = 11/15 (73%), Positives = 12/15 (80%), Gaps = 0/15 (0%)

```
P 15   3   LGGGGGAGGGGLVGG  17
           +GGGGG GGGG  GG
Sbjct  3   IGGGGGGGGGGSCGG  17
```

Score = 27.5 bits (55), Expect = 0.005,Identities = 14/27 (52%), Positives = 15/27 (56%), Gaps = 6/27 (22%)

```
P 15   1   GLLGGGGGAGG------GGLVGGLLNG  21
```

```
          G+ GGGGG GG       GG  GG  NG
Sbjct  2   GIGGGGGGGGGGSCGGQGGGCGGCSNG  28
```

> DBAASP_2873|3113|Acanthoscurrin-2 from *Acanthoscurria gomesiana* [Tarantula spider], Antibacterial (Gram-), Antifungal, Target: *E.coli* SBS363, *C.albicans*
Length=130, Score = 32.7 bits (67),Expect = 3e-004,Identities = 15/21 (71%), Positives = 15/21 (71%), Gaps = 0/21 (0%)

```
P 15   1   GLLGGGGGAGGGGLVGGLLNG  21
           G LGGG G GGGGL GG L G
Sbjct  34  GGLGGGKGLGGGGLGGGGLGG  54
```

> DBAASP_2872|3112|Acanthoscurrin-1 from *Acanthoscurria gomesiana* [Tarantula spider], Antibacterial (Gram-), Antifungal, Target: *E.coli* SBS363, *C.albicans*
Length=132, Score = 32.7 bits (67),Expect = 3e-004, Identities = 15/21 (71%), Positives = 15/21 (71%), Gaps = 0/21 (0%)

```
P 15   1   GLLGGGGGAGGGGLVGGLLNG  21
           G LGGG G GGGGL GG L G
Sbjct  60  GGLGGGKGLGGGGLGGGGLGG  80
```

>APD_453|AP01405|Leptoglycin from *Leptodactylus pentadactylus*,Antibacterial(Gram-)
Length=22,Score = 29.7 bits (60),Expect = 7e-004,Identities = 13/18 (72%), Positives = 14/18 (78%), Gaps = 3/18 (17%)

```
P 15   1   GLLG---GGGGAGGGGLV  15
           GLLG   GGGG GGGGL+
Sbjct  5   GLLGPLLGGGGGGGGGLL  22
```

Score = 26.7 bits (53), Expect = 0.008,Identities = 15/25 (60%), Positives = 15/25 (60%), Gaps = 9/25 (36%)

```
P 15   1   GLLGG------GGGAGGGGLVGGLL  19
           GLLGG      GGG GGG   GGLL
Sbjct  1   GLLGGLLGPLLGGGGGGG---GGLL  22
```

PEPTIDE 16. MGGLLGGVNGGGKGGGGPGAP

>APD_453|AP01405|Leptoglycin from *Leptodactylus pentadactylus*,Antibacterial(Gram-)
Length=22,Score = 30.6 bits (62),Expect = 4e-004,Identities = 12/17 (71%), Positives = 14/17 (82%), Gaps = 0/17 (0%)

```
P 16   1   MGGLLGGVNGGGKGGGG  17
           +GGLLG + GGG GGGG
Sbjct  3   LGGLLGPLLGGGGGGGG  19
```

> CAMPSQ3913 Ctenidin-1 from *Cupiennius salei* [Wandering spider], Antimicrobial
Length=119, Score = 30.6 bits (62),Expect = 0.002,Identities = 13/18 (72%), Positives = 13/18 (72%), Gaps = 0/18 (0%)

```
P 16   2    GGLLGGVNGGGKGGGGPG  19
            GGL GG  GGG GGGG G
Sbjct  90   GGLGGGRGGGGYGGGGGG  107
```
